# Ischemic Etiology and Clinical Outcomes Following Cardiac Resynchronization Therapy

**DOI:** 10.3390/medicina56030110

**Published:** 2020-03-04

**Authors:** Teruhiko Imamura

**Affiliations:** Second Department of Medicine, University of Toyama, 2630 Sugitani Toyama, Toyama 930-0194, Japan; teimamu@med.u-toyama.ac.jp; Tel.: +81-76-434-2281

**Keywords:** hemodynamics, heart failure, myocardial infarction

## Abstract

Optimal patient selection for cardiac resynchronization therapy is crucial. There are several concerns that allow to better clarify the association between the ischemic etiology of heart failure and the response to cardiac resynchronization therapy. The type of ischemic coronary disease has an impact on the responses to cardiac resynchronization therapy. The prognostic impact of cardiac resynchronization therapy on cardiac death including heart transplantation and durable ventricular assist device implantation is another concern.

Dear Editor,

Basinskas and colleagues demonstrated that the ischemic etiology of heart failure was an independent predictor of mortality following cardiac resynchronization therapy [1]. Given the cost-effectiveness and urgency of heart failure, optimal patient selection for cardiac resynchronization therapy should be essential [2]. There are two concerns that should improve the implication of their findings. 

First, more detailed data about the types of ischemia would speak to the mechanism of why ischemic etiology was associated with higher mortality. When dominant causes of ischemia are associated with myocardial infarction, it would explain the reduced achievement of cardiac reverse remodeling during cardiac resynchronization therapy. When most cases involved ischemia of the right ventricle, it would explain the impairment of right ventricular function with a lower tricuspid annular plane excursion. 

Second, many patients with advanced heart failure have recently received cardiac replacement therapy including heart transplantation or ventricular assist device [3]. It would be better to clarify whether these therapeutic outcomes were censored, counted as mortality as cardiac death, or not performed in their cohort. 
